# RoBuST: an integrated genomics resource for the root and bulb crop families Apiaceae and Alliaceae

**DOI:** 10.1186/1471-2229-10-161

**Published:** 2010-08-06

**Authors:** Ashwini Bhasi, Doug Senalik, Philipp W Simon, Brajendra Kumar, Vinu Manikandan, Philge Philip, Periannan Senapathy

**Affiliations:** 1 Genome International Corporation, 8000 Excelsior Drive, Suite 202, Madison, Wisconsin 53717, USA; 2 United States Department of Agriculture, Agricultural Research Service, Vegetable Crops Research Unit, Department of Horticulture, University of Wisconsin, Madison, Wisconsin 53706, USA; 3 International Center for Advanced Genomics and Proteomics, 83, 1st Cross Street, Nehru Nagar, Kottivakkam, Chennai 600096, India

## Abstract

**Background:**

Root and bulb vegetables (RBV) include carrots, celeriac (root celery), parsnips (Apiaceae), onions, garlic, and leek (Alliaceae)—food crops grown globally and consumed worldwide. Few data analysis platforms are currently available where data collection, annotation and integration initiatives are focused on RBV plant groups. Scientists working on RBV include breeders, geneticists, taxonomists, plant pathologists, and plant physiologists who use genomic data for a wide range of activities including the development of molecular genetic maps, delineation of taxonomic relationships, and investigation of molecular aspects of gene expression in biochemical pathways and disease responses. With genomic data coming from such diverse areas of plant science, availability of a community resource focused on these RBV data types would be of great interest to this scientific community.

**Description:**

The RoBuST database has been developed to initiate a platform for collecting and organizing genomic information useful for RBV researchers. The current release of RoBuST contains genomics data for 294 Alliaceae and 816 Apiaceae plant species and has the following features: (1) comprehensive sequence annotations of 3663 genes 5959 RNAs, 22,723 ESTs and 11,438 regulatory sequence elements from Apiaceae and Alliaceae plant families; (2) graphical tools for visualization and analysis of sequence data; (3) access to traits, biosynthetic pathways, genetic linkage maps and molecular taxonomy data associated with Alliaceae and Apiaceae plants; and (4) comprehensive plant splice signal repository of 659,369 splice signals collected from 6015 plant species for comparative analysis of plant splicing patterns.

**Conclusions:**

RoBuST, available at http://robust.genome.com, provides an integrated platform for researchers to effortlessly explore and analyze genomic data associated with root and bulb vegetables.

## Background

In the present decade, rapid advancements in DNA sequencing and molecular genetics techniques have generated a wealth of plant genomics, genetics, and breeding data. Whole genome sequences of several plants (arabidopsis, rice, wheat, grapes, etc) are now available and the sequencing of a number of additional plants is ongoing (lotus, papaya, cassava, tomato, potato, etc). The rapid proliferation of plant genome annotations generated by these efforts has led to the development of several data analysis platforms focused on specific plant groups - Gramene for grass genomics [1], SOL Genomics Network (SGN) for solanaceae [2], Legume Information System for legume species [3], GrainGenes for Triticeae and Avena [4] and GDR genome database for Rosaceae [5] are some examples. These resources integrate genomic sequence data, genetic maps, and phenotypic data associated with specific plant groups into a single user friendly platform and encourage researchers to get involved in annotation efforts. Such integrated repositories of plant group-specific data with active community contributions can greatly aid researchers in gaining a deeper understanding of plant genetics, plant taxonomy, plant pathology and plant physiology, and are valuable tools for supporting research initiatives focused on addressing challenges confronted in agriculture.

Interestingly, most of the plant data analysis platforms available today are focused on plant groups with high economic value and are associated with data collection from genome sequencing projects. Very few data analysis tools are available for less-studied "minor" and "orphan" crops with no ongoing genome sequencing efforts. Integrated data collection and annotation platforms focused on specific orphan crop families would be invaluable for researchers working on these crops.

Root and bulb vegetables (RBV) represent one such group of orphan crops [6,7]. These include carrots, celeriac (root celery), parsnips (Apiaceae), onions, garlic, and leek (Alliaceae, sometimes referred to as the Allioideae subtribe Amaryllidaceae) - food crops common to virtually all agricultural regions and consumers' plates around the world. Yet the community of plant scientists working on RBV crops is small, perhaps as small as 10 to 25 full-time equivalent scientists in academic and government programs worldwide devoted to each of these crops. Most of this effort comes from breeders, geneticists, taxonomists, plant pathologists, and plant physiologists. A similar level of effort also comes from seed companies worldwide [8,9]. Yet even with a relatively small scientific community, genomic data is becoming increasingly more abundant, primarily from efforts to develop molecular genetic maps, delineate taxonomic relationships and investigate molecular aspects of gene expression in biochemical pathways and disease response. With genomic data coming from such diverse areas of plant science, availability of a database focused on these root and bulb vegetable crop data would be of great interest to this small scientific community.

Plants that form storage roots and bulbs are included in several web sites that have a taxonomical or regional focus. For example, turnip and rutabaga are included in the Eurpoean Brassica Database [10], yacon (*Smallanthus sonchifolius*), a member of the Asteraceae, and wild yam (*Dioscorea villosa*), a member of the Dioscoraceae, are included in the Tropical Plant Database [11]. Databases of the latter type rarely include genomic information. Furthermore, databases such as the SOL Genomics Network [2] include tuber-bearing species and genomic data for this plant family but comparable databases that focus on storage root or bulb-forming plants have not been developed. While the Apiaceae and Alliaceae include many species that do not form storage roots or bulbs, many wild and domesticated species in these two plant families do.

The RoBuST database has been developed for initiating a platform to collect and organize genomic information useful for genetics, breeding, taxonomy, plant physiology and other data highly relevant to root and bulb vegetables. The current release of RoBuST provides a comprehensive collection of sequence annotations of genes, RNAs, proteins, splice signals, regulatory elements and ESTs belonging to two root and bulb orphan crop families—Alliaceae and Apiaceae. RoBuST also has a unique data collection of Alliaceae/Apiaceae-specific gene function and trait annotations, molecular taxonomy data, genetic linkage maps, markers and biosynthetic pathways as well as an exhaustive collection of plant splicing data. A variety of sequence analysis and visualization tools are available in the RoBuST web interface for the integrated analysis of these diverse data sets.

## Construction and content

### Database and web interface development

The RoBuST database was developed in MySQL 4.1.The RoBuST web interface was developed using the CGI.pm, DBI.pm and GD.pm modules of Perl (5.8.8), and runs on an Apache (2.0.53) web server. Graphical display of genes, RNAs, intron-exon structures, splice junctions, and alternative splicing patterns were implemented in RoBuST using Gene Plot, Splice Signal Plot and AS Plot modules from the EuSplice [12] project. Additional graphical modules to display regulatory elements and other sequence elements were developed specifically for RoBuST using GD.pm.

### Extraction of sequence annotations

Sequence records of Alliaceae and Apiaceae plant families were downloaded from GenBank [13] by submitting batch queries to its Entrez web interface. Gene, mRNA and other sub-sequence annotations are presented in a pre-determined data structure under the Feature Table section of GenBank records with each sub-sequence elements described as Feature Keys [14]. We carefully analyzed the Feature Table structure and developed Perl programs to parse the Feature key data format and efficiently extract sub-sequence datasets. The Perl programs extracted position co-ordinates, sequences and additional annotations associated with each of following sequence data types: (1) Gene, (2) RNA, (3) CDS (4) EST, (5) Promoter, (6) Regulatory elements (Signal Peptide, Replication Origin, 5'UTR, Enhancer, TATA Signal, CAAT Signal, -10 Signal, -35 Signal, PolyA Signal, PolyA Site, Protein Bind, 3'UTR), (7) Repeat elements (Repeat Region, Repeat Unit, Transposons) and (8) Miscellaneous elements (Misc Feature, Precursor RNA, Prim Transcript, Mature Peptide, Primer Bind, Misc Signal, Stem Loop, Variation, Misc RNA, Transit Peptide). The extracted sequence and annotation data were populated into the back-end MySQL database.

### Extraction of plant splicing data

Plant-specific annotation data files ("gbpln*.seq.gz") were downloaded from GenBank and all GenBank gene records which belong to plant species from Kingdom Viridiplantae were extracted. Since we were interested in splice signal information, all single-exon gene records were removed from this data set. This left 348,081 GenBank records covering 6015 Viridiplantae plant species, each of which had multi-exon gene annotations. These were further parsed to extract position coordinates and sequences associated with gene, mRNA, exon and intron structure, and donor and acceptor splice signals.

### Implementing Ontologies

We submitted seventeen new carrot and garlic trait terms to Trait Ontology (TO), which is part of the Plant Ontology Consortium [15]. All seventeen were accepted into TO and were assigned TO IDs and parent-child relationships. We implemented a hierarchy-based display of TO term lineage for these traits in the RoBuST Trait Ontology viewer using GraphViz [16].

We successfully mapped Gene Ontology (GO) [17] function annotations to 119 RoBuST genes as follows: InterPro [18] IDs corresponding to RoBuST genes were extracted from the database cross references (db_xrefs) provided under the CDS feature key tag in their corresponding GenBank files. The GO annotation file "interpro2go" was downloaded from InterPro and InterPro IDs associated with each of the 119 genes were mapped to the gene ontology information available in this file.

## Utility and Discussion

### Sequence Annotations

RoBuST provides a comprehensive platform for integrated visual analysis of various types of sequence annotations from 294 Alliaceae and 816 Apiaceae plant species. This includes genes, mRNAs, tRNAs, rRNAs, ESTs, splice signals, promoters, regulatory elements, signal peptides, transposons, molecular taxonomy sequences and several additional types of sequence elements (Table 1). Users can access sequence annotations of interest using keyword or ID-based search options available in the web interface.

**Table 1:** Distribution of sequence data types in RoBuST

Sequence Data Type	Alliaceae	Apiaceae
Structural genes	931	2732
mRNA/CDS	372	2082
Promoters	1	25
Transposons	6	56
EST	20201	2522
tRNA	99	384
rRNA	770	2252
5'UTR	23	28
Enhancer	-	10
TATA signal	1	26
CAAT signal	-	12
-10 signal	-	1
-35 signal	-	1
PolyA signal	33	37
PolyA site	14	60
Protein bind	-	2
3'UTR	35	26
Repeat region	108	573
Signal peptide	26	13
Replication origin	-	1
Misc feature	1250	1426
Precursor RNA	-	6
Prim transcript	1	10
Mature peptide	28	22
Primer bind	62	5
Misc signal	-	2
Stem loop	-	1
Variation	-	15
Misc RNA	262	4818
Transit peptide	-	5
Mitochondrail genes	15	60
Plastid genes	328	2006

Numbers of entries for major categories of Alliaceae and Apiaceae sequences are indicated.

**Figure 1:**
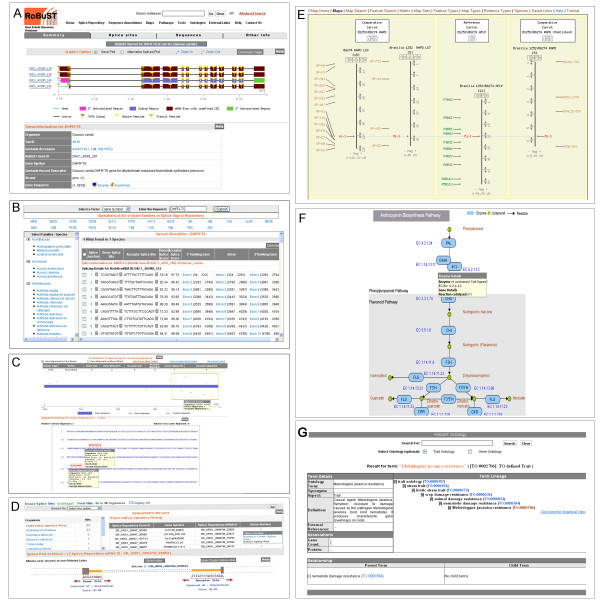
**Screenshots of the RoBuST web interface**. **A**, graphical gene detail plot in RobuST. Moving the mouse over introns or exons activates a text box with information on that component, and links are provided to detailed splice plots, alternative splice plots, gene sequence, and other information. **B**, plant splice repository. Taxonomic limits can be placed on information retrieved, and sequence information and splice plots can be retrieved for individual elements listed. **C**, SpliceBlast-PL. This tool allows identification of potential intron/exon structures within a submitted sequence, and provides links and information about the matching splice sequence**. D**, SpliceMatch-PL. This module allows data mining to find splice signal information from the RoBuST database. **E**, CMap viewer. Individual linkage groups or markers may be selected and examined in closer detail, and linkage groups with markers in common can be viewed together. **F**, Biosynthetic pathway page. Moving the mouse over individual enzymes or compounds will activate a text box with details and links to further information, including gene details. **G**, Trait Ontology viewer. Parent and child terms are displayed, with links to details about the ontology terms.

Gene and RNA sequence annotations can be visualized as graphical plots in RoBuST's results page. The graphical plots allow users to zoom in and efficiently analyze exons, introns, splice signal sequences, splice junctions as well as translated and untranslated regions using color-coded display and pop-up options, and integrate all related information in one place without browsing away from the main result page. Users can also view the alternative transcription initiation (ATI), alternative splicing (AS), and alternative transcription termination (ATT) events in genes with multiple transcript isoforms. The graphical plots also display regulatory elements such as promoters, polyA, mature peptides, signal peptides, and all other sequence element types found within the gene or RNA sequence (Figure 1A).

The results page also has several informational tabs which provide additional data on the gene or RNA of interest. The "Summary" tab provides general information on the sequence record such as gene/RNA name and description, organism name, GenBank Accession/ID and links to the original GenBank record. The "Sequences" tab, provides options to download sequences of gene, RNA, coding sequence (CDS), protein and other sequence annotations (promoters, ESTs, regulatory elements, repeat elements etc). If a gene has an exon-intron structure, then the splice signal sequences of each of its splice junctions can be viewed by clicking on the "Splice Sites" tab. Database cross-references to five independent external databases (UniProtKB [19], InterPro, GOA [20], HSSP [21], and PDB [22] related to the gene/RNA of interest can be accessed through the "Other Info" tab.

### Sequence Analysis Tools

RoBuST supports the diverse sequence analysis requirements of Alliaceae/Apiaceae research community by integrating the following sequence analysis tools to its user-friendly web interface. (1) Customized access to plant family-specific and species-specific BLAST [23] searches; (2) Primer Design Tools: Primer3Plus [24] and ExonPrimer3—a customized version of Primer3 which we developed so that users can design specific primers for exons from any Alliaceae or Apiaceae gene; (3) Emboss [25] pair-wise sequence alignment tools: *Water *for local alignment and *Needle *for global alignment; (4) Emboss open reading frame detection tool: *Getorf*; (5) VecScreen [26] for detecting vector contamination; (6) Splign [27] and Spidey [28] for exon-intron structure detection and splicing analysis.

### Plant Splice Repository (PSR)

**Figure 2:**
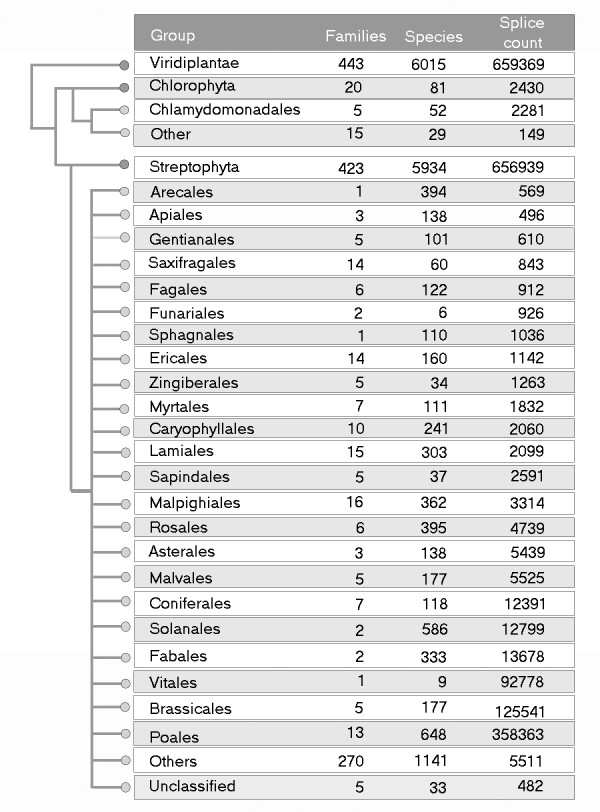
**A summary of the phylogenetic distribution of splice signal data content in the RoBuST Plant Splice Repository (PSR)**.

A comprehensive collection of annotated plant splice signals can be a valuable tool for analyzing splicing mechanisms in plants. For this purpose, we have created the Plant Splice Repository (PSR)—unique collection of plant-specific splicing data associated with 6015 plants species belonging to 443 plant families extracted from GenBank plant sequence records. PSR currently contains 659,369 donor and acceptor splice signal pairs (Figure 2) and is a valuable resource for comparison and analysis of splicing patterns in plants.

PSR can be mined to retrieve splicing annotations associated with an entire plant family, a specific plant species or even a specific gene of interest (Figure 1B). For any given gene, users can view its exon-intron structure, analyze donor and acceptor splice signals, exon and intron sequences flanking splice junctions as well as access the distribution of putative splice signals surrounding annotated splice junctions. Users can also download these gene-specific PSR datasets for further analysis.

PSR also has two unique splicing analysis tools which support in-depth mining of the extensive splicing data content. *SpliceBlast-PL *(Figure 1C) identifies potential plant-specific exon-intron structures within a query sequence of interest by aligning the query sequence against the entire plant splice signal collection in PSR and *SpliceMatch-PL *(Figure 1D) can be used to identify the prevalence of a splice signal sequence of interest within various plant genomes. It accepts user-submitted splice signal sequences as a query and retrieves hits in different plant species which have this same splice signal. Each of these hits can be analyzed in detail using graphical splice signal plots.

### Genetic Linkage Maps

Linkage maps play an important role in plant genetics and genomics and are highly valuable resources for the genome analysis of plant species which do not have whole genome sequencing data and hence do not have any physical maps available. Genetic linkage maps can be updated when maps from several studies are consolidated. While several genetic linkage maps are available for different Alliaceae and Apiaceae species, they are spread across various publications or are maintained as an internal collection by research groups for their own specific analysis and hence are not available in one place where external users can access and analyze them. Our effort in RoBuST is to create a centralized repository for all Alliaceae and Apiaceae genetic linkage maps, each of which are linked to information on its specific mapped markers. As part of that effort, we have currently added 12 linkage maps: 8 for carrot, 3 for garlic and 1 for onion into RoBuST (Table 2). These maps can be accessed and viewed through the cMap viewer [29] deployed on the RoBuST server. Users can browse through genetic maps, view marker data, and search for specific markers of interest. Graphical comparison between different genetic maps can also be performed and this functionality can be used to generate a single comparative view of multiple maps of interest, to analyze common markers and to conduct comparative analysis of the loci of interest (Figure 1E). We encourage and invite members of the Alliaceae and Apiaceae research community to submit additional linkage maps and annotations and be active participants in developing a complete collection of linkage map data for these two plant families.

**Table 2:** Complete and partial genetic linkage maps in RoBuST

Species	Map Set	Mapping Population Size	Population Type	Number of Linkage Groups	Markers
*Daucus carota*	Brasilia 1252 × B6274 RAPD Linkage Map (Leonardo Boiteux; partial map for linkage group bearing *Mj-1 *only)	442	F2	1 (partial)	5
	Brasilia 1252 × B6274 AFLP Linkage Map (Leonardo Boiteux; partial map for linkage group bearing *Mj-1 *only)	188	F2	1 (partial)	11
	Brasilia 1252 × B6274 RAPD Linkage Map (Leonardo Boiteux)	412	F2	24	111
	B493 × QAL Linkage Map (Carlos Santos)	183	F2	18	216
	B493 × QAL Linkage Map with DcMaster markers (Dariusz Grzebelus)	159	F2	18	311
	B493 × QAL Linkage Map with carotenoid genes (Brian Just)	183	F2	18	256
	BSB × HCM Linkage Map (Carlos Santos)	160	F2	18	245
	9304X7262 Linkage Map (B.S. Vivek)	103	F2	11	113
					
*Allium sativum*	Garlic Linkage Map 1 (Meryem Ipek)	53	F1	20	181
	Garlic Linkage Map 2 (Meryem Ipek)	53	F1	13	104
	Garlic Linkage Map 3 (Y. Zewdie)	84	S1	9	37
					
*Allium cepa*	Brigham Yellow Globe 15-23 × Alisa Craig 43 (Michael J. Havey)	59	F2	8	209

Number of linkage groups and markers for *Daucus *and *Allium *maps are listed.

### Biosynthetic Pathways

RoBuST provides detailed graphical displays of the carotenoid and anthocyanin biosynthetic pathways of *Daucus carota *and S-alk(en)yl cysteine sulfoxide and allium flavor chemistry pathways of *Allium sativum *(Figure 1F). Information on pathway steps, substrates, enzymes catalyzing the reaction, links to the RoBuST records of pathway genes, genetic linkage map details for these genes, and 2D molecular structure of the pathway elements is available in the pathway display page. We plan to incorporate additional pathways from Alliaceae/Apiaceae species and welcome submissions from the research community to enhance the current collection.

### Plant Taxonomy and Systematics

DNA sequences have become a standard tool used by plant taxonomists [30]. Internal Transcribed Spacer (ITS) is often the first sequence used to evaluate and classify plants by species, but a widening array of genic regions is also being used. It is becoming recognized that a single sequence does not discriminate accurately among species of a genus, so sequence information from several different regions are now becoming widely accepted as markers for taxonomic classification in plants. Since there is a growing need for the Alliaceae and Apiaceae research community to have direct access to family-specific molecular taxonomy sequences, we have parsed and extracted phylogenetic markers (e.g., ITS1 & ITS2, Non Transcribed Spacer (NTS), External Transcribed Spacer (ETS)) and "barcoding" genes (e.g., matK) associated with Apiaceae and Alliaceae families from GenBank sequence data. Table 3 gives the distribution of some common molecular taxonomy sequences in RoBuST. This collection of 9837 molecular taxonomy sequences is a valuable reference resource for intergeneric/intra-generic and interspecies/intra-species level taxonomy analysis and classification efforts.

**Table 3:** Common molecular taxonomy sequence annotations in RoBuST

Marker Class	Number of Apiaceae species with this sequence	Total number of sequences for Apiaceae	Number of Alliaceae species with this sequences	Total number of sequences for Alliaceae
**External Transcribed Spacer (ETS)**	2	2	45	47
**Internal Transcribed Spacer (ITS)**	1475	4844	319	1203
**Non Transcribed Spacer (NTS)**	419	791	86	146
**matK**	7	7	-	-
**rp116-rp114 spacers**	3	3	-	-
**nad7**	-	-	2	2
**18S rRNA**	137	224	51	61
**5.8S rRNA**	926	1689	318	601
**26S rRNA**	136	216	1	1

Numbers of species and sequences for major marker classes included in molecular taxonomic studies of Alliaceae and Apiaceae are listed.

### Trait Ontology and Gene Ontology Browsers

We have implemented an ontology browser for exploring functional annotations and trait features of Alliaceae and Apiaceae plants. Gene Ontology (GO) functional annotations for 43 Alliaceae and 76 Apiaceae genes can be accessed and analyzed in the GO annotation browser in RoBuST (Figure 1G).

For traits, we used the well-established Trait Ontology (TO) collection for defining carrot and garlic trait terms. Each carrot trait which matched existing TO trait terms was mapped to their specific TO ID and TO annotation. Since several carrot and garlic trait definitions were not available in TO, we submitted these new trait definitions to Trait Ontology. This resulted in the addition of 17 new trait terms to the TO collection (Table 4).

**Table 4:** Trait ontology terms accepted by TO consortium

TO ID	Trait Ontology Term
TO:0002694	Flavor trait
TO:0002695	Beta-carotene content
TO:0002696	Alpha carotene content
TO:0002697	Cyclic carotene content
TO:0002698	Acyclic carotene content
TO:0002699	Lycopene content
TO:0002700	Pungency
TO:0002701	Lutein content
TO:0002702	Cercospora leaf spot resistance
TO:0002703	Protist disease resistance
TO:0002704	Meloidogyne incognita resistance
TO:0002706	Meloidogyne javanica resistance
TO:0002707	Petiole color
TO:0002708	Xylem color
TO:0002709	Phloem color
TO:0002710	Root shape
TO:0002711	Total water soluble content

Trait ontology terms added for Alliaceae and Apiaceae are listed.

Trait and functional annotations can be queried through the ontology browser using general keyword searches or by specific searches using ontology terms or accession IDs. Users can view complete information for each ontology term as well as parent and child terms and their relationships (Figure 1G).

### Future Directions

We plan to expand the current data coverage in RoBuST in the future, and develop it into a one-stop resource for the root and bulb research community by providing comprehensive coverage of genomic data for all available root and bulb plant species. Since comparative analysis of genomic data from tuber forming plants and root and bulb plants could prove useful for understanding common mechanisms of bulb and tuber formation, we plan to link genomic information on tuber-bearing species from SGN to RoBuST. We will also incorporate interactive tools that can be used by root and bulb researchers to annotate sequences, phenotypes and traits, and to submit linkage maps, markers, molecular phylogeny data, pathways and other relevant data types which are of interest to the user community. Additionally, several comparative genomics tools will be made available in the next release of RoBuST and these tools will allow user-friendly cross-species comparative analysis of sequence annotations not only within the root and bulb plant group, but also against well annotated genomes such as rice, wheat, arabidopsis, barley, grape, potato and tomato. Since inter-database comparative analysis would be very useful in plant genome analysis and knowledge discovery, we will implement link integration of RoBuST data to Gramene and Ensembl. We will also provide gateways to ExDom [31] a database resource that will enable the comparative functional analysis of proteins and domains from these genomes. Furthermore, we will enable the capability for analyzing mutations in genes, RNAs and regulatory sequences in RoBuST using the EuSplice resource [12] and comparative analysis of alternative splicing events using the AspAlt resource [32].

Since the current sequence data in RoBuST is from a single data source—GenBank, we did not have data heterogeneity issues in this version release. However, we will be faced with data heterogeneity when we expand the RoBuST content to include data from different databases like SGN, Gramene and Ensembl in the next version release of RoBuST. To address this, we will adapt DAS protocols [33] in RoBuST and set up a DAS server which will make the import and export of data easy. This will ensure that RoBuST's future development and updates are not complicated by diverse data formats and data heterogeneity from different data sources.

## Conclusion

The plant research community in agriculture often has a commodity-specific focus. Consequently, the availability of detailed information from diverse scientific disciplines has long been of interest to crop scientists. As genomic data is the common language of all biological disciplines, a focused collection of genomic data for an individual plant species or family provides a valuable tool for researchers specializing in particular crops. This fact led to the development of GrainGenes, SolGenes, etc. To this end, RoBuST has been developed for plant scientists working on root and bulb vegetable crops. The array of genomic information on these relatively poorly studied plants is not extensive but even today over 47,000 GenBank sequence records have been generated for Apiaceae and Alliaceae alone. With the recent advancements in next generation sequencing technology, an increase in genomic data for root and bulb orphan crops in public databases is imminent. Furthermore, there is little doubt that the whole genome of one or more species in Apiaceae will be sequenced in the next few years, especially since many species in this family have genomes in the size range of rice and tomato. The extraordinarily large size of Alliaceae genomes will impede their whole genome sequencing projects, but that fact notwithstanding, much more sequence information of Alliaceae will certainly be generated. With the certainty that next-generation sequencing will become a common research tool, the management of these large sequence datasets is receiving much attention by genomicists. Discussion of next-generation plant genetics is already underway [34] and similar strategies are being planned for use in plant taxonomy and physiology. We anticipate that specialized databases like RoBuST will become even more valuable for crop specialists as the database grows. While RoBuST assembles tools useful for genomics of root and bulb vegetables, it also provides tools to tie genomic data to genetic linkage data, plant traits and biochemical pathways of interest to geneticists, taxonomists, and physiologists, and it creates links to other related databases. This collection of features provides a network for communication from the DNA sequence level to the trait and crop level with relative ease. The tools assembled in RoBuST are intended to be of wide immediate interest to researchers working on root and bulb crops, and to also serve as a model of assembling diverse comparable data useful for any crop.

## Availability and requirements

RoBuST is freely available to all non-commercial users at http://robust.genome.com.

## Authors' contributions

AB designed the database and web interface, integrated the data and wrote the manuscript. DS and PSW provided annotated data, defined key functionalities of the resource and helped write the manuscript. BK, VM and PP worked on development and implementation of the database and web interface. PS and PWS conceived the project, AB coordinated it. PS participated in project design and critically revised the manuscript. All authors read and approved the final manuscript.

## References

[B1] Gramene http://www.gramene.org/

[B2] Sol Genomics network http://solgenomics.net

[B3] Legume Information System http://www.comparative-legumes.org

[B4] GrainGenes: A Database for Triticeae and Avena http://wheat.pw.usda.gov/GG2

[B5] GDR Genome database for Rosaceae http://www.rosaceae.org

[B6] Havey MJ Application of Genomic Technologies to Crop Plants: Opportunities and Challenges Crop Sci 2004 44 1893 1895 10.2135/cropsci2004.1893a

[B7] Nelson R Naylor RL Jahn MM The role of genomics research in improvement of "orphan" crops Crop Sci 2004 44 1901 1904 10.2135/cropsci2004.1901

[B8] Brooks HJ Vest G Public programs on genetics and breeding of horticultural crops in the United States HortScience 1985 20 826 830

[B9] Frey KJ National Plant Breeding Study-I. Special Report 98 1996 Iowa State University, Ames

[B10] European Brassica Database http://documents.plant.wur.nl/cgn/pgr/brasedb/

[B11] Tropical Plant Database http://www.rain-tree.com/yacon.htm

[B12] Bhasi A Pandey RV Utharasamy SP Senapathy P EuSplice: a unified resource for the analysis of splice signals and alternative splicing in eukaryotic genes Bioinformatics 2007 23 1815 1823 10.1093/bioinformatics/btm084 17344236

[B13] Benson DA Karsch-Mizrachi I Lipman DJ Ostell J Sayers EW GenBank Nucleic Acids Res 2009 37 D26 D31 10.1093/nar/gkn723 18940867 PMC2686462

[B14] The DDBJ/EMBL/GenBank Feature Table Definition http://www.ncbi.nlm.nih.gov/collab/FT/

[B15] Bruskiewich R Coe EH Jaiswal P McCouch S Polacco M Stein L Vincent L Ware D The Plant Ontology Consortium and plant ontologies Comp Funct Genomics 2002 3 137 142 10.1002/cfg.154 18628842 PMC2447263

[B16] GraphViz http://search.cpan.org/dist/GraphViz

[B17] Ashburner M Ball CA Blake JA Botstein D Butler H Cherry JM Davis AP Dolinski K Dwight SS Eppig JT Harris MA Hill DP Issel-Tarver L Kasarskis A Lewis S Matese JC Richardson JE Ringwald M Rubin GM Sherlock G Gene ontology: tool for the unification of biology. The Gene Ontology Consortium Nat Genet 2000 25 25 29 10.1038/75556 10802651 PMC3037419

[B18] Hunter S Apweiler R Attwood TK Bairoch A Bateman A Binns D Bork P Das U Daugherty L Duquenne L Finn RD Gough Julian Haft D Hulo N Kahn D Kelly E Laugraud A Letunic I Lonsdale D Lopez R Madera M Maslen J McAnulla C McDowall J Mistry J Mitchell A Mulder N Natale D Orengo C Quinn AF InterPro: the integrative protein signature database Nucleic Acids Res 2009 37 D211 D215 10.1093/nar/gkn785 18940856 PMC2686546

[B19] Schneider M Lane L Boutet E Lieberherr D Tognolli M Bougueleret L Bairoch A The UniProtKB/Swiss-Prot knowledgebase and its Plant Proteome Annotation Program J Proteomics 2009 72 567 573 10.1016/j.jprot.2008.11.010 19084081 PMC2689360

[B20] Barrell D Dimmer E Huntley RP Binns D O'Donovan C Apweiler R The GOA database in 2009—an integrated Gene Ontology Annotation resource Nucleic Acids Res 2009 37 D396 D403 10.1093/nar/gkn803 18957448 PMC2686469

[B21] Dodge C Schneider R Sander C The HSSP database of protein structure-sequence alignments and family profiles Nucleic Acids Res 1998 26 313 315 10.1093/nar/26.1.313 9399862 PMC147243

[B22] Sussman JL Lin D Jiang J Manning NO Prilusky J Ritter O Abola EE Protein Data Bank (PDB): database of three-dimensional structural information of biological macromolecules Acta Crystallogr D Biol Crystallogr 1998 54 1078 1084 10.1107/S0907444998009378 10089483

[B23] Altschul SF Gish W Miller W Myers EW Lipman DJ Basic local alignment search tool J Mol Biol 1990 215 403 410 2231712 10.1016/S0022-2836(05)80360-2

[B24] Untergasser A Nijveen H Rao X Bisseling T Geurts R Leunissen JA Primer3Plus, an enhanced web interface to Primer3 Nucleic Acids Res 2007 35 W71 4 10.1093/nar/gkm306 17485472 PMC1933133

[B25] Rice P Longden I Bleasby A EMBOSS: the European Molecular Biology Open Software Suite Trends Genet 2000 16 276 277 10.1016/S0168-9525(00)02024-2 10827456

[B26] VecScreen http://www.ncbi.nlm.nih.gov/VecScreen/VecScreen.html

[B27] Kapustin Y Souvorov A Tatusova T Lipman D Splign: algorithm for computing spliced alignments with identification of paralogs Biol Direct 2008 3 20 10.1186/1745-6150-3-20 18495041 PMC2440734

[B28] Spidey http://www.ncbi.nlm.nih.gov/IEB/Research/Ostell/Spidey/

[B29] Youens-Clark K Faga B Yap IV Stein L Ware D CMap 1.01: a comparative mapping application for the Internet Bioinformatics 2009 25 3040 3042 10.1093/bioinformatics/btp458 19648141 PMC2773250

[B30] Álvarez I Wendel JF Ribosomal ITS sequences and plant phylogenetic inference Mol Phylogenet and Evol 2003 29 417 434 10.1016/S1055-7903(03)00208-2 14615184

[B31] Bhasi A Philip P Manikandan V Senapathy P ExDom: an integrated database for comparative analysis of the exon-intron structures of protein domains in eukaryotes Nucleic Acids Res 2009 37 D703 D711 10.1093/nar/gkn746 18984624 PMC2686582

[B32] Bhasi A Philip P Sreedharan VT Senapathy P AspAlt: A tool for inter-database, inter-genomic and user-specific comparative analysis of alternative transcription and alternative splicing in 46 eukaryotes Genomics 2009 94 48 54 10.1016/j.ygeno.2009.02.006 19285128

[B33] Dowell RD Jokerst RM Day A Eddy SR Stein L The Distributed Annotation System BMC Bioinformatics 2001 2 7 10.1186/1471-2105-2-7 11667947 PMC58584

[B34] Nordborg M Weigel D Next-generation genetics in plants Nature 2008 456 720 723 10.1038/nature07629 19079047

